# LncRNA EPB41L4A-AS2 represses Nasopharyngeal Carcinoma Metastasis by binding to YBX1 in the Nucleus and Sponging MiR-107 in the Cytoplasm

**DOI:** 10.7150/ijbs.55557

**Published:** 2021-05-11

**Authors:** Mingyu Du, Xinyu Hu, Xuesong Jiang, Li Yin, Jie Chen, Jing Wen, Yanxin Fan, Fanyu Peng, Luxi Qian, Jing Wu, Xia He

**Affiliations:** 1 Jiangsu Cancer Hospital, The Affiliated Cancer Hospital of Nanjing Medical University, Jiangsu Institue of Cancer Research, Nanjing, China.; 2 The Fourth Clinical School of Nanjing Medical University, Nanjing, China.

**Keywords:** EPB41L4A-AS2, nasopharyngeal carcinoma, YBX1, EMT, miR-107, LATS2

## Abstract

Nasopharyngeal carcinoma (NPC) is known for its potential to progress to the lymph nodes and distant metastases at an early stage. As an important regulator in tumorigenesis biological processes, the functions of lncRNA in NPC tumor development remain largely unclear. In this research, the expression of EPB41L4A-AS2 in NPC tissues and cells was analyzed via real-time quantitative polymerase chain reaction (qRT-PCR). CCK8, colony formation, and EDU experiments were used to determine the viability of NPC cells. Transwell and wound healing assays were performed to test NPC cell migration and invasion. RNA pull-down and mass spectrometry analysis were used to identify potential binding proteins. Then, a popliteal lymph node metastasis model was established to test NPC metastasis. EPB41L4A-AS2 is repressed by transforming growth factor-beta, which is downregulated in NPC cells and tissue. It is associated with the presence of distant metastasis and adverse outcomes. The univariate and multivariate survival assays confirmed that EPB41L4A-AS2 expression was an independent predictor of progression-free survival (PFS) in patients with NPC. Biological analyses showed that overexpression of EPB41L4A-AS2 reduced the metastasis and invasion of NPC *in vitro* and *in vivo*, but had no significant effect on cell proliferation. Mechanistically, in the nucleus we identified that EPB41L4A-AS2 relies on binding to YBX1 to reduce the stability of Snail mRNA to enhance the expression of E-cadherin and reverse the progression of epithelial-to-mesenchymal transition (EMT). In the cytoplasm, we found that EPB41L4A-AS2 blocked the invasion and migration of NPC cells by promoting LATS2 expression via sponging miR-107. In a whole, the findings of this study help to further understand the metastasis mechanism of NPC and could help in the prevention and treatment of NPC metastasis.

## Introduction

Nasopharyngeal carcinoma (NPC) is a head and neck squamous cell carcinoma (HNSCC) that arises from the epithelium of the nasopharynx. The incidence of NPC is 25 per 100,000 in southern China and South-East Asia. However, NPC is rare in the rest of the world, demonstrating its unique geographical concentration 1. Some epidemiological evidence supports that the occurrence and development of NPC is multifactorial, and is closely associated with genetic susceptibility, racial predisposition, family aggregation, Epstein-Barr virus (EBV) infection, unhealthy dietary habits, and adverse environmental conditions 2, 3. NPC has some unique characteristics: (1) 95% of the pathological types are non-keratinizing undifferentiated carcinoma; (2) it has an early tendency to spread to the local parapharyngeal space, and more than 70% of NPC patients have swelling of the lymph nodes in the neck at the initial presentation. NPC is considered to be more invasive and display higher metastatic behavior than other head, and neck malignant tumors; (3) due to the deep location and distinctive anatomical structure of the nasopharynx and sensitivity to radiotherapy, the standard treatment modality for NPC is concurrent chemoradiotherapy (CCRT) with cisplatin-based regimens 4. Unfortunately, relapse and distant metastases often occur.

Long non-coding (Lnc)RNAs are a type of non-coding ribonucleic acid (RNA) that is larger than 200 nucleotides 5. Extensive research shows that it participates in a number of physiological and pathological biological processes. Recently, lncRNA was found to be involved in tumorigenesis biological processes, including the carcinogenic or suppressive mechanism, and can even be used as a cancer biomarker 6, 7. The sense strand, also known as the coding strand, is the strand in a gene that has the same base sequence as the transcribed product mRNA (except T for U). The strand complementary to the sense strand in a gene is called the antisense strand or template strand. This is a template that plays a role in transcription. Antisense lncRNA is a lncRNA classification. Antisense strand transcripts can interfere with deoxyribonucleic acid (DNA) transcription or the stability of mRNA and are hybridized with a coding strand or antisense strand transcripts 8. EPB41L4A-AS2 is a novel antisense lncRNA. The correlated mRNA expression patterns and functions of EPB41L4A-AS2 in NPC should be further explored.

Tumor invasion and metastasis are partly attributed to epithelial-to- mesenchymal transition (EMT), characterized by decreased expression of epithelial proteins and increased expression of mesenchymal proteins 9, 10. The morphological feature of EMT is from the shape of epithelial cells to the appearance of spindle-shaped fibroblasts, while the functional feature is the loss of cell adhesion and the potential for cell migration and invasion. Molecularly, this is the decrease in epithelial markers (such as E-cadherin) and ascension of mesenchymal markers (such as Vimentin and N-cadherin) 11. Various extracellular signaling molecules promote EMT through a series of cascade reactions, and transforming growth factor-beta (TGF-β) is the main moderator of EMT in cancer cells 12. Moreover, transcription factors, such as snail, can act as the main moderator of EMT by activating the mesenchymal phenotype and inhibiting the epithelial phenotype 13.

The Y-box binding protein 1(YBX-1) belongs to a member of the cold-shock domain protein superfamily, and has an evolutionarily conserved cold-shock domain (CSD) 14. As a well-known DNA/RNA binding protein, YBX1 has been reported involved in some lncRNAs perform their biological functions during the tumor development and progression. For example, Zhang et al. reported that lncRNA HOXC-AS3 promotes tumorigenesis of gastric cancer via binding to YBX1 15. In this study, we explored the relationship between lncRNA EPB41L4A-AS2 and YBX1 in the NPC metastasis. In the cytoplasm, lncRNAs usually serve as the sponges of miRNAs to release the expressions of miRNAs-mediated targets. Among these lncRNA-related miRNAs, miR-107 was famous for its important roles in many cancers progression 16. Han et al reported that lncRNA MIR503HG reversed the tumorigenesis of cervical cancer via repressing the expression of miR-107 17, while another report showed lncRNA LINC00467 contributes to tumorigenesis of cervical cancer cells through targeting miR-107 18. However, weather lncRNA EPB41L4A-AS2 sponged with miR-107 in NPC development was deserved our further investigation.

In this work, our study demonstrates the anti-metastatic roles of EPB41L4A-AS2 in NPC and identifies the specific factors responsible for metastasis.

## Materials and Method

### Tissue specimens

Sixty-five NPC biopsy samples and 10 normal human nasopharyngeal epithelial tissue samples were obtained from Jiangsu Cancer Hospital. The NPC samples were confirmed by three experienced pathologists. Each patient signed an informed consent form. This research was approved by the Ethics Review Committee of our center.

### Cell lines and cell cultures

Under standard conditions (37 °C, 5% CO_2_), human NPC cell lines , such as CNE-1, CNE-2, 5-8 F, 6-10B and SUNE1 cells, were grown in RPMI 1640 medium (Invitrogen, USA) covered with 10% fetal bovine serum (FBS) ( Gibco, USA), while another cell line NP69, known as the human immortalized nasopharyngeal epithelial, was cultured in the serum-free medium (Invitrogen, USA) containing bovine pituitary extract (BD, USA).

### Cell transfection

LncRNA Smart Silencer specific to EPB41L4A-AS2 and Smart Silencer NC were designed and compounded by RiboBio (Guangzhou, China). Plasmid pcDNA3.1-EPB41L4A-AS2 was purchased from Genecreate (Wuhan, China). SUNE1 and CNE2 were grown to 70% confluence in a 6-well plate and transfected with Smart Silencer (100 nmol) or plasmid (5 µg) using Lipofectamine 2000 (Invitrogen). Furthermore, 24-48 h after transfection, the knockdown, and overexpression efficacy were evaluated by RT-PCR.

### qRT-PCR analyses

Total RNA isolation from the NPC tissue and cells was performed with TRIzol reagent (Invitrogen,USA). Nanodrop 2000 (Invitrogen,USA) was used to measure the quality and concentration of the RNA, and then reverse transcribed into cDNA. The qRT-PCR analysis was executed by a PrimeScript^TM^ RT Reagent Kit (Takara). The mixed reagents were carried out on an ABI7300 real-time PCR machine (Applied Biosystems,USA).The primer pairs were EPB41L4A -AS2: 5′-GTCGCAGTTAGGGGAGACAC-3′ and 5′-TGGCTACCCAGCTAACA AGC-3′; β-actin: 5′-GGACTTCGAGCAAGAGATGG-3′ and 5′-AGCACTGTGTTGGCGTACAG-3′; U6: 5′-CTCGCTTCGGCAGCA-CA-3′ and 5′-AACGCTTCACGAATTTGCGT-3′. β-actin and U6 were used as endogenous controls. The relative expressions were calculated using the 2 ^-ΔΔCT^ method.

### Western blot analysis

Total protein was collected from SUNE1 and CNE2 using radioimmunoprecipitation (RIPA) containing PMSF (Beyotime, China). Sodium dodecyl sulfate-polyacrylamide gel (SDS-PAGE) was used to separate proteins. During electrophoresis, the proteins were transferred from the gel to a polyvinylidene difluoride (PVDF) membrane. The PVDF membranes were blocked for 15 min in a rapid blocking solution (Beyotime, China) and immunoblotted with the primary antibodies overnight at 4 °C, including anti-YBX1 (Abcam,USA), anti-E-cadherin (CST,USA), anti-Vimentin (CST,USA), anti-N-cadherin (CST,USA), anti-Snail (CST,USA), and anti-β-actin (CST,USA). The next day, the PVDF membrane and the secondary antibody were incubated for 2 h at room temperature. Immune signals were visualized using enhanced chemiluminescent detection reagent (Beyotime, Shanghai, China). Finally, immunoreactive bands were detected by ChemiDoc XRS+ (Bio-Rad, USA).

### Transwell migration, invasion, and wound‑healing assays

Functional experiments were performed in biological duplicates, as previously described 19.

### Cell cycle assay

According to the disparate DNA contents, the cell cycle distribution was divided into G0/G1, S, G2, and M phases. Briefly, SUNE1 and CNE2 were collected and fixed in 70% pre-cold ethanol and then stained with propidium iodide (BD, USA). The cell cycle was analyzed using FACS Vantage SE (BD, USA).

### EDU

The 5-Ethynyl -2'- deoxyuridine (EDU) assay was implemented with the EDU kit (RiboBio, China). In short, cells were transfected with smart silencer specific to EPB41L4A-AS2, EPB41L4A-AS2 overexpressing plasmid and their corresponding negtive controls, and then seeded into 96-well plates (5×10^5^ cells/well). Cells were fixed in 4% polyformaldehyde and then infiltrated with 0.5% Triton X-100. After washing the cells 3 times, 100 µL of Apollo® mixture was added, and subsequently, 100 µL of Hoechst33342 was stained. Finally, the photograph was taken using a fluorescent microscope.

### mRNA stability

Transfected NPC cells were treated with 5 mM of actinomycin D or dimethyl sulfoxide (DMSO) and cultured at the indicated time points 20.

### Subcellular fractionation location

Nucleus and cytoplasmic RNA from SUNE1 and CNE2 were separated using the PARIS™ Kit (Life Technologies, USA). The expression levels of EPB41L4A-AS2, U6, and S18 were used as controls for the nucleus and cytoplasm, respectively. The results were measured using RT-qPCR.

### Fluorescence *in situ* hybridization

The FISH experiment was implemented with the Fluorescent *in situ* Hybridization Kit (Genepharma, China). The FISH probe of LncRNA EPB41L4A-AS2 was obtained from Genepharma (Shanghai, China). After the cell slide, cells were fixed and hybridized with 10 μl of probe storage solution and 80 μl of hybridization buffer mixture following the manufacturer's instructions. Finally, the results were observed using a fluorescence microscope.

### RNA pulldown assay

In brief, 3-terminal biotin-labeled RNA EPB41L4A-AS2 and its control probe were transfected into cells using the Biotin RNA Labeling Mix (Ambion). SUNE1 cells, together with streptavidin magnetic beads (Life Technologies, USA) were incubated for 24 h. The EPB41L4A-AS2-interacting proteins were separated by SDS-PAGE. Afterward, the gels were silver-dyed, and the retrieved proteins were uncovered by mass spectrometry (MS) or western blotting.

### RNA immunoprecipitation (RIP)

RIP assays were performed according to the instructions of the EZMagna RIP kit (Millipore, USA). Briefly, cell suspension was added to RIP buffer was supplemented with magnetic beads conjugated to YBX1 (CST) and rabbit immunoglobulin G (IgG) antibody (Abcam). The concentration of the co-precipitated RNAs that were adsorbed onto magnetic beads was measured with a NanoDrop spectrophotometer. The expression of isolated immunoprecipitated RNA was determined using real-time PCR analysis.

### Animal experiment

BALB/c nude mice that were 6-8 weeks old were acquired from Yangzhou University Medical Center (Yangzhou, China). Ten nude mice were randomly divided into experimental and control groups. 20μL of SUNE1-vector or SUNE1-EPB41L4A-AS2 cells (3×10^6^) expressing green fluorescent protein (GFP) were planted into the footpads of the mice. GFP was used to determine mice that had popliteal lymph node metastasis. After 6 weeks, the mice were sacrificed, and the tumors were weighed after autopsy. Hereafter, popliteal lymph nodes were enucleated. Sections of the lymph nodes were subjected to paraffin embedding and underwent hematoxylin and eosin (H&E) staining for pathology verification. The expression of EMT-associated genes (E-cadherin, vimentin, N-cadherin, and snail) in the metastatic lymph nodes was determined by immunohistochemical (IHC) analysis. All animal experiments were authorized by the Animal Science Committee of the Animal Science of Nanjing, China.

### Dual Luciferase Reporter Assays

Dual luciferase experiments were as described in our previous article 21.

### Statistical analysis

Data were analyzed using Prism 6 (GraphPad, USA). The data from the research are presented as the mean ± SD. The student's t-test or a one-way analysis of variance (ANOVA) were employed to determine the differential expression between groups. Overall survival (OS) and progression-free survival (PFS) were calculated by the Kaplan-Meier method. P-values of <0.05 and <0.001 were identified as statistically significant. The research was performed independently in triplicate.

## Results

### EPB41L4A-AS2 is regulated by the transforming growth factor-beta (TGF-β) signaling pathway

**Figure 1:**
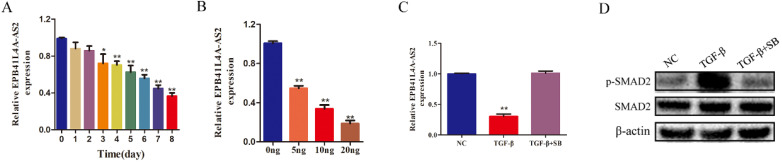
** EPB41L4A-AS2 was downregulated by TGF-β in NPC cells.** (A) TGF-β stimulation inhibited the expression of EPB41L4A-AS2, depending on the exposure time. (B) TGF-β stimulation repressed the expression of EPB41L4A-AS2, depending on the concentration. (C and D) Expression of phosphorylated SMAD2 and SMAD2 and relative expression of EPB41L4A-AS2 by western blot or RT-qPCR. *P < 0.05 and **P < 0.01.

In a previous study, we found that EPB41L4A-AS2 was attenuated by TGF-β 22. As shown in Figures 1A and 1B, TGF-β was found to repress EPB41L4A-AS2 in a time- and dose-dependent manner. To determine the underlying mechanisms by which TGF-β regulates the expression of EPB41L4A-AS2, we employed the TGF-β receptor inhibitor, SB431542 23, which has been reported to effectively reduce the phosphorylation of SMAD2, to block the activity of TGF-β pathway. As shown in Figure 1C and 1D, SB43152 reversed the repression of EPB41L4A-AS2 induced by TGF-β. It is known that TGF-β consists of classic SMAD pathways and non-SMAD pathways 24. Notably, SMAD2 phosphorylation is a vital step in activating the TGF-β SMAD signaling pathway. We also detected the effects of EPB41L4A-AS2 knockdown on the expressions of SMAD2 and p-SMAD2. The results showed that EPB41L4A-AS2 cannot influenced SMAD2 or p-SMAD2 expressions (Supplementary Figure 1). Our data provide evidence that EPB4AL4A-AS2, as the downstream effector of the TGF-β signaling pathway, was regulated through the classic SMAD signaling pathway.

### EPB41L4A-AS2 was downregulated in NPC and was associated with a definitive diagnosis

**Figure 2:**
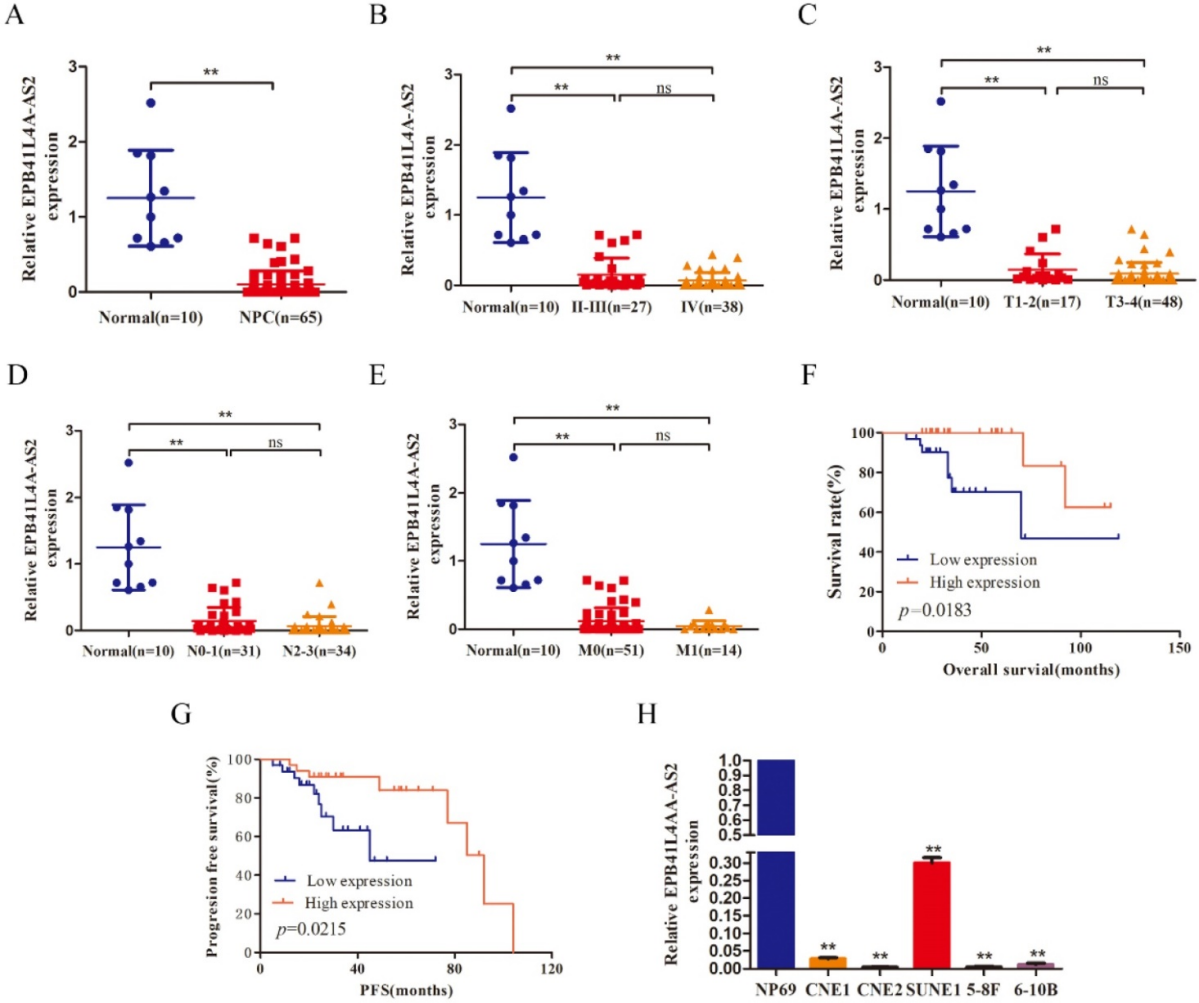
** lncRNA EPB41L4A-AS2 was downregulated in NPC tissues and cells.** (A) qRT-PCR assays were used to test the expression levels of EPB41L4A-AS2 in 10 normal nasopharyngeal and 65 NPC tissue samples. (B) The expression of EPB41L4A-AS2 was assayed in normal nasopharyngeal tissues and patients with different TNM stages. (C) The expression of EPB41L4A-AS2 was assayed in normal nasopharyngeal tissues and different primary tumors of patients. (D) The expression of EPB41L4A-AS2 was assayed in normal nasopharyngeal tissues and different lymph node metastasis tissues. (E) The expression of EPB41L4A-AS2 was assayed in normal nasopharyngeal tissues and different metastasis tissues. (F) The Kaplan-Meier curve analysis of the impact of EPB41L4A-AS2 expression on overall survival (OS) of patients with NPC. (G) Kaplan-Meier curve analysis of the impact of EPB41L4A-AS2 expression on progression-free survival (PFS) of patients with NPC. (H) The expression level of EPB41L4A-AS2 in NP69 and NPC cells was determined by qRT-PCR. *P < 0.05 and **P < 0.01. ns: not significant.

To uncover the level of EPB41L4A-AS2 expression in NPC, qRT-PCR was used to detect mRNA levels in 65 NPC tumor tissues, 10 normal nasopharyngeal tissues, and several NPC cell lines, as well as human immortalized nasopharyngeal carcinoma cell lines (NP69). The data exhibited that the expression level of EPB41L4A-AS2 in NPC tissue was reduced, compared with normal tissues (Figure 2A). In line with this, compared with NP69, EPB41L4A-AS2 in NPC cell lines was also decreased (Figure 2H). The correlation between EPB41L4A-AS2 expression and the characteristics analyzed revealed that lower EPB41L4A-AS2 was remarkably correlated with TNM stage (Figure 2B and 2C), lymph node metastasis (Figure 2D), and distant metastasis (Figure 2E). Pearson's correlation analysis indicated that EPB41L4A-AS2 expression was positively associated with the presence of lymph node metastasis, but showed no significant correlations with age, gender, TNM stage, and T stage (Table 1). Interestingly, it appeared that EPB41L4A-AS2 expression might be linked with the presence of distant metastasis in patients with NPC. The survival analysis demonstrated that a higher EPB41L4A-AS2 expression was linked with poorer PFS and OS (Fig 2F and G). In addition, the univariate and multivariate survival assays confirmed that EPB41L4A-AS2 expression was an independent predictor of PFS in patients with NPC (Tables 2 and 3). However, regarding OS, more data is required to ascertain whether EPB41L4A-AS2 is an independent prognostic factor in NPC patients. Therefore, it appears that EPB4AL4A-AS2 might function as a tumor suppressor in NPC metastasis.

### EPB41L4A-AS2 influenced NPC metastasis and invasion *in vitro* and *in vivo*

**Figure 3:**
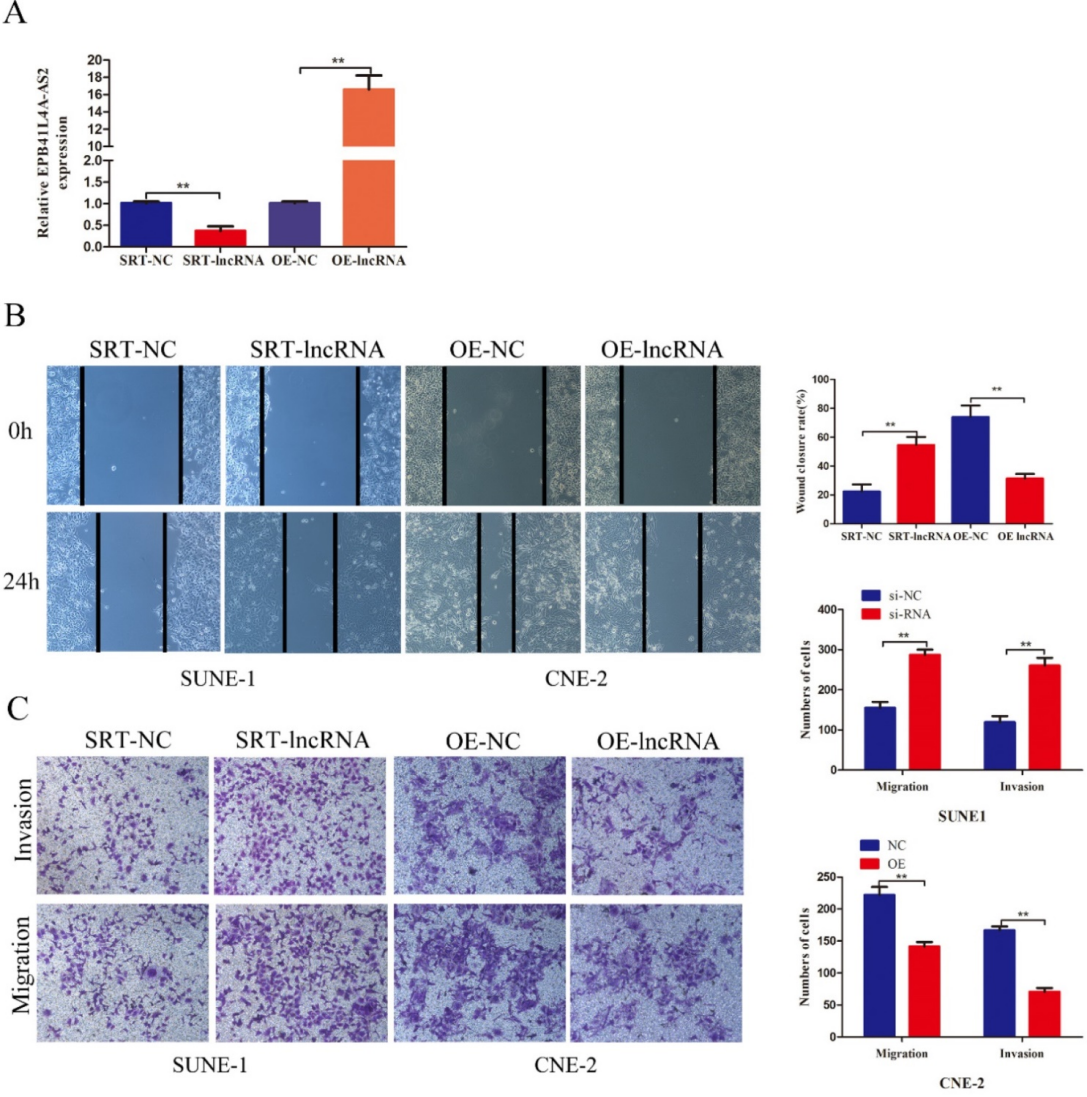
** EPB41L4A-AS2 reduced the invasion and migration of NPC cells.** (A) The efficiencies of the interference and overexpression of EPB41L4A-AS2 were detected via qRT-PCR. (B and C) Transwell invasion, and migration assays indicated that EPB41L4A-AS2 reduced the NPC cells ability to invade and migrate. *P < 0.05 and **P < 0.01.

RT-qPCR showed that EPB41L4A-AS2 effectively interfered and overexpressed (Figure 3A). Both loss- and gain-of-function experiments were used to survey the role of EPB41L4A-AS2 on cell migration, invasion ability, and proliferation using transwell, colony formation, CCK8, and EDU assays. Compared with the control group, there was no evident change in the cell proliferation function (Supplementary Figures 2A and 2B). Similar results were observed for EDU (Supplementary Figure 2C). On the other hand, the cell cycle and apoptosis analysis of CNE2 and SUNE1 cells after transfection with SRT-lncRNA or OE-lncRNA by flow cytometry (FCM) analysis found no significant differences (Supplementary Figure 2D). However, the results revealed that the silencing of EBP41L4-AS2 occurred in accelerated cell migration and invasion, while re-expression of EBP41L4-AS2 had the opposite effect (Figure 3B and 3C). Thus, the functions of EPB41L4A-AS2 were mostly centered on the cell invasion and migration of NPC cells.

**Figure 4:**
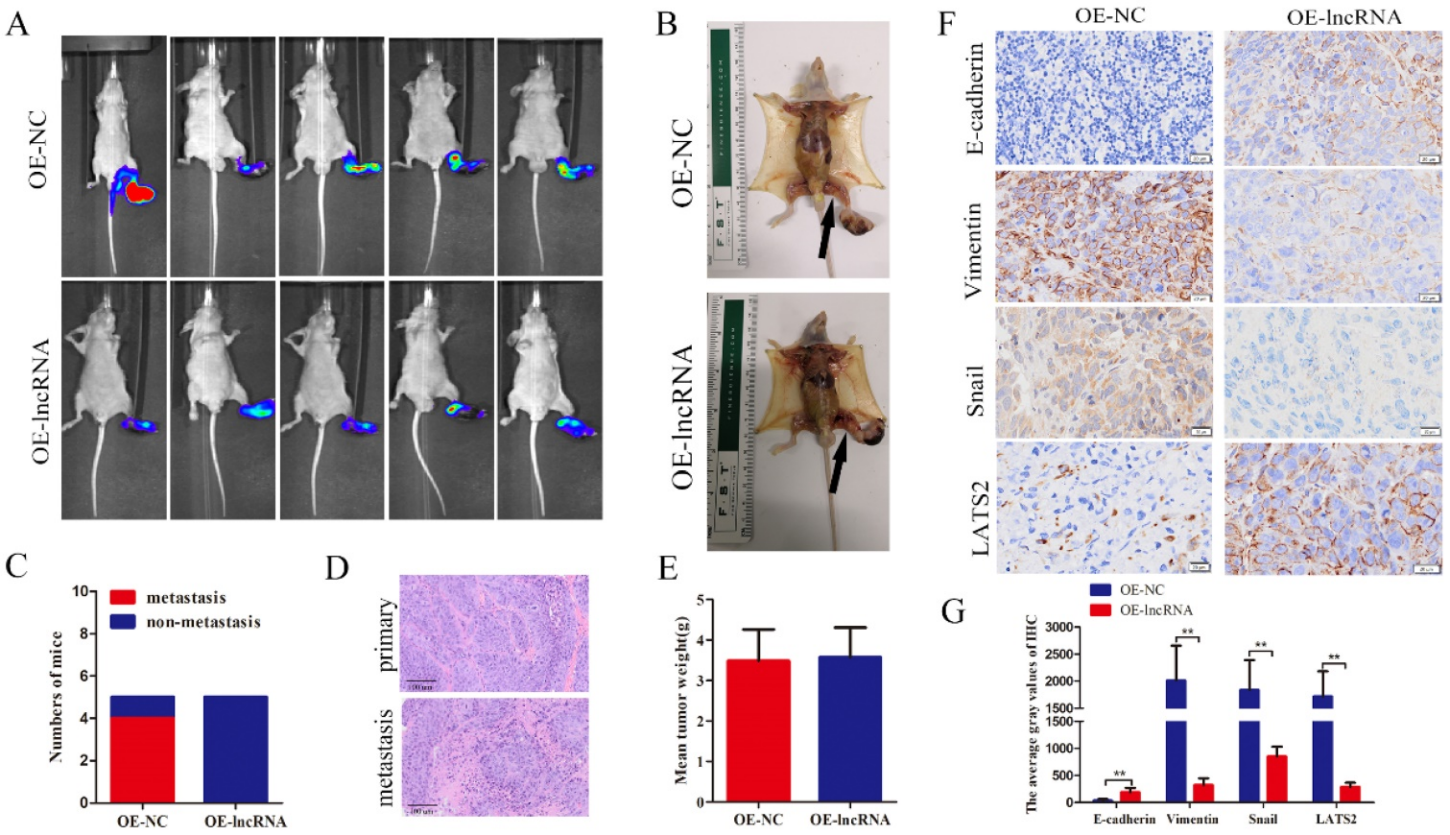
** EPB41L4A-AS2 suppressed metastasis *in vivo*.** (A) The metastatic popliteal lymph nodes were assessed under a laser microscope. (B) The presence of the metastatic popliteal lymph nodes was pictured after dissection. The arrow indicates the metastatic popliteal lymph node. (C) The incidences of popliteal lymph node metastasis of each group were counted. (D) H&E staining showed that the tumor was cancerous. (E) The data indicated that overexpression of EPB41L4A-AS2 had little effect on the average tumor weight. (F and G) IHC was used to assess the expression of E-cadherin, Vimentin, Snail and LATS2 in tumors. *P < 0.05 and **P < 0.01.

To further investigate whether EPB41L4A-AS2 inhibits NPC metastasis *in vivo*, a model of spontaneous lymph node metastasis in nude mice with EPB41L4A-AS2 overexpressing NPC cells was established. As shown in Figure 4A-C, the number of mice with lymph node metastases in the EPB41L4A-AS2 overexpression groups was less than that in the control groups. HE staining certified that the primary and metastatic foci are tumor tissues (Figure 4D). Not surprisingly, overexpression of EPB41L4A-AS2 exerted little effect on the growth of NPC *in vivo* (Figure 4E), which was consistent with the findings of our functional experiments. In addition, IHC was applied to assess the expression of E-cadherin, vimentin, snail and LATS2 in tumors. Consistent with the above results, compared with the corresponding control group, E-cadherin and LATS2 expression were distinctly increased, whereas vimentin and snail subsided (Figure 4F and 4G). These above results confirm that EPB41L4A-AS2 inhibits NPC migration and invasion *in vitro* and *in vivo*.

### EPB41L4A-AS2 directly bound to YBX1

**Figure 5:**
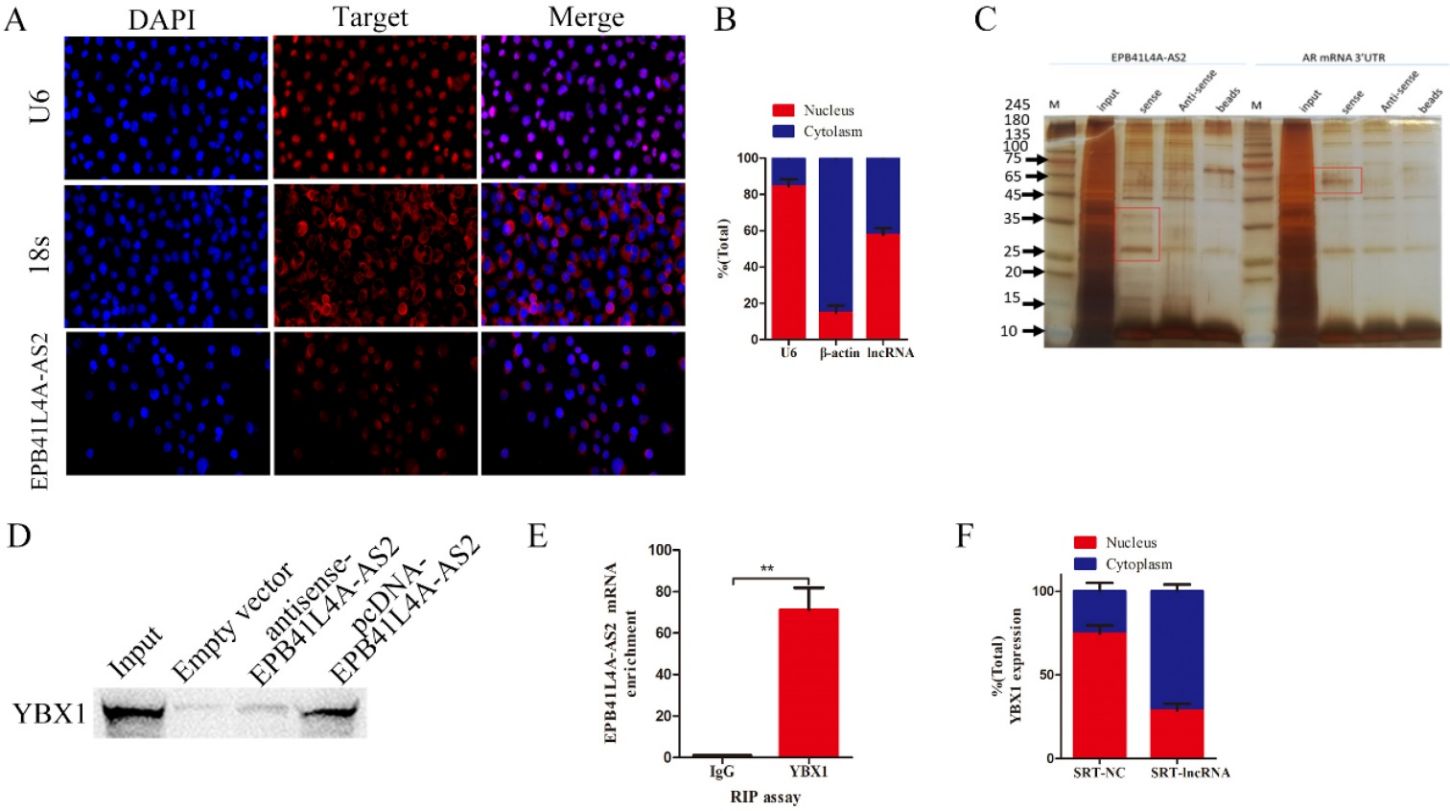
** EPB41L4A-AS2 directly binding to YBX1.** (A) FISH assays were applied to determine the subcellular localization of EPB41L4A-AS2 in NPC. U6 was mainly located in the nucleus, while 18S was mainly located in the cytoplasm. (B) Nuclear mass separation assays demonstrated that EPB41L4A-AS2 was located both in the nucleus and cytoplasm. (C).SDS-PAGE gel was used to explore the EPB41L4A-AS2 specific binding protein gels (D) YBX1 was identified by SDS-PAGE. (E) RIP assays confirmed that EPB41L4A-AS2 directly binds to YBX1. (F) Subcellular location of YBX1 in SUNE1 cells. *P < 0.05 and **P < 0.01.

To explore the precise molecular mechanisms and process of EPB41L4A-AS2 regulating NPC metastasis, we first analyzed the subcellular localization of EPB41L4A-AS2 using FISH and subcellular fractionation location assays. Subcellular localization results indicated that it was both distributed in the nucleus and cytoplasm (Figures 5A and 5B), suggesting that EPB41L4A-AS2 is likely to perform its functions at the transcription level and post-transcription. As is known that the regulation of downstream genes by lncRNA binding protein is the most common mode of action that lncRNA performs in the nucleus 25-27. We carried out RNA pull-down experiments to examine EPB41L4A-AS2-associated protein and performed a mass spectrometry analysis of the binding proteins (Figures 5C-5D). Among them, YBX1 attracted the attention of our research group, due to its well-known tumor-promoting role in many cancers. Our previous work showed that LINC01133 inhibited the EMT, cell invasion, and metastasis of NPC by directly binding to YBX1 28. We also analyzed whether EBP41L4-AS2 can directly bind to YBX1 using RIP experiments, and the results supported our hypothesis (Figure 5E). Due to the reason that YBX1 not only expressed in the cytoplasm, but also in the nucleus, we made further investigation to explore the potential mechanism that EPB41LRA-AS2 influenced YBX1 in NPC cells. The subcellular fractionation location assays exhibited that EPB41L4A-AS2 knockdown enhanced the enrichment of YBX1 in the cytoplasm, which indicated that EPB41L4A-AS2 affected the location of YBX1 via binding to YBX1 (Figure 5F). Collectively, in the nucleus, EPB41L4A-AS2 performed its functions, partly binding to YBX1 to affect downstream effectors.

### Biological function of EPB41L4A-AS2 depended on the binding of YBX1

**Figure 6:**
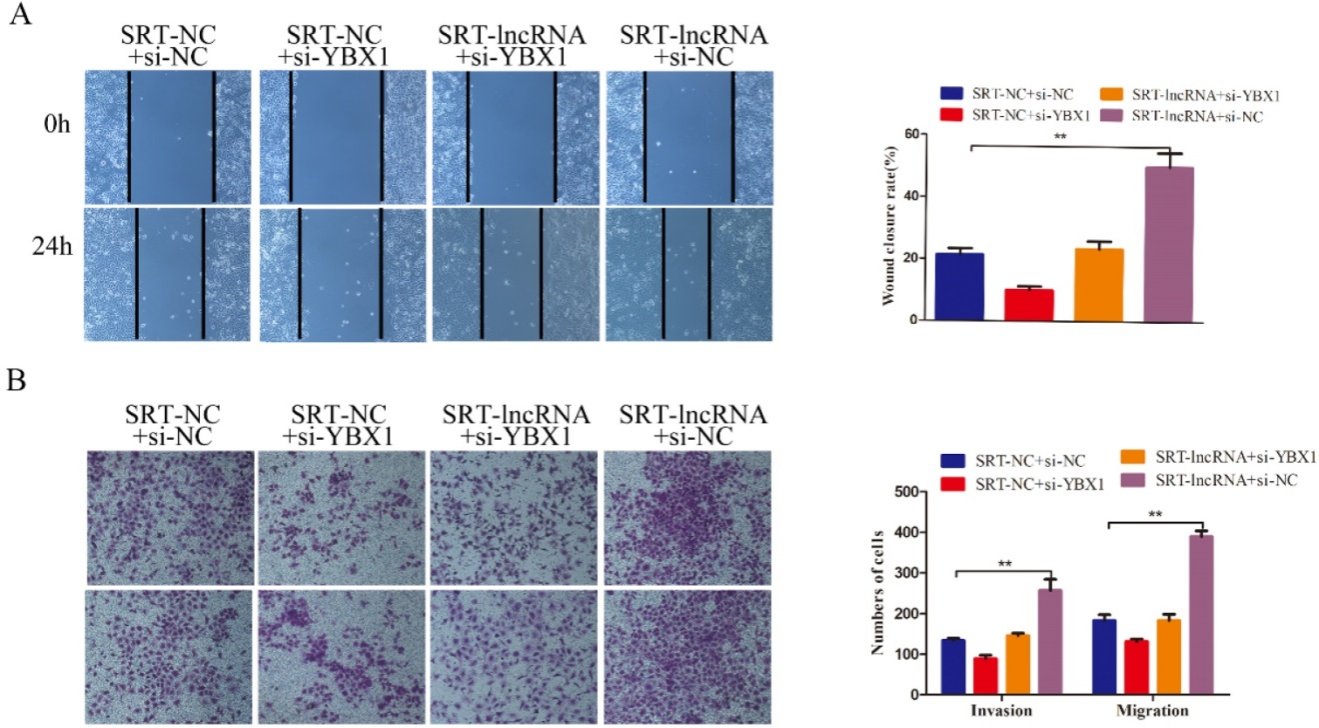
** EPB41L4A-AS2 repressed the invasion and migration of NPC cells via the binding of YBX1.** (A) Wound healing assays were performed to determine the migration of NPC cells. (B) The invasion and migration abilities of NPC cells were detected by transwell assays under different treatments. *P < 0.05 and **P < 0.01.

Additionally, we continued to observe the co-transfection changes in the tumor-promoting effect of diminished EPB41L4A-AS2 with either YBX1-siRNA or control-siRNA in SUNE1 cells. The siRNA against YBX1 effectively reduced YBX1 expressions in NPC cells (Supplementary Figure 3). Knocking down YBX1 partially reversed the invasion and migration potential of EPB41L4A-AS2 depletion (Figures 6A and 6B). These results confirm that EPB41L4A-AS2 can inhibit NPC tumor invasion and metastasis through YBX1.

### EPB41L4A-AS2 regulates snail mRNA stability via YBX1

**Figure 7:**
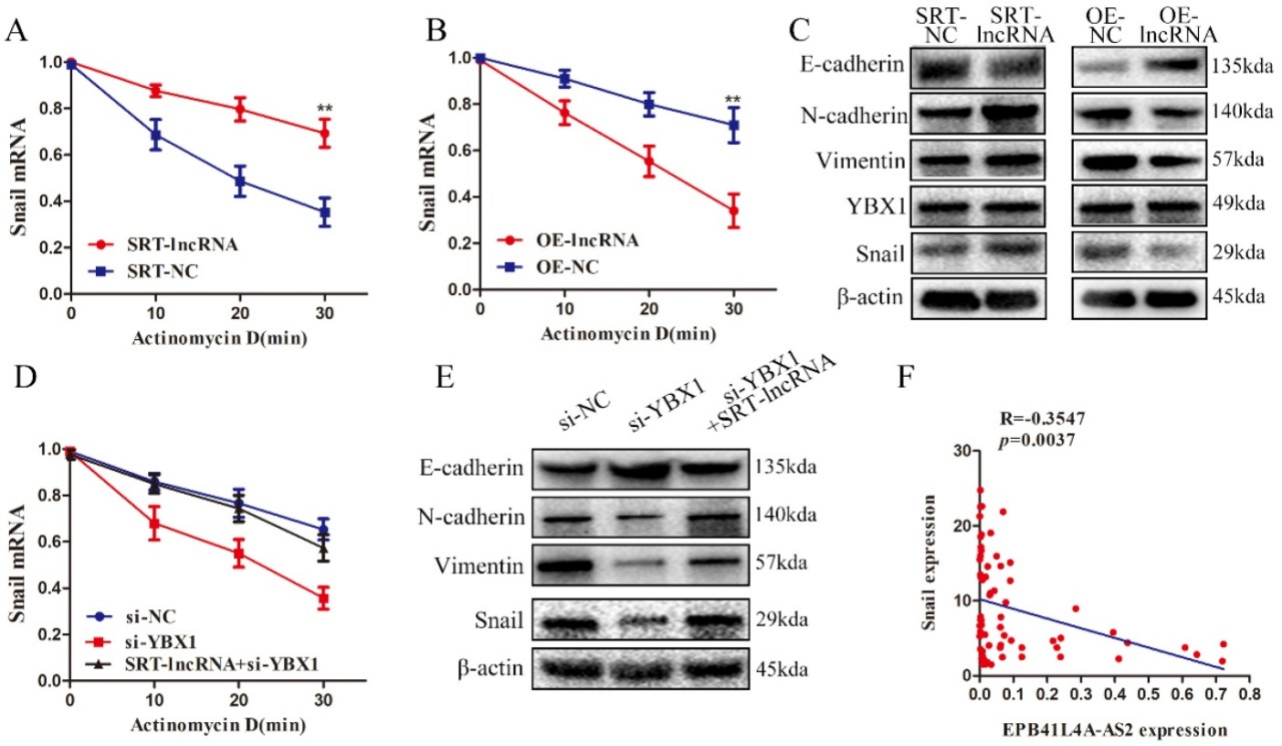
** EPB41L4A-AS2 binds YBX1 to regulate snail mRNA stability.** (A and B) The change in Snail half-life after down-regulating or up-regulating EPB41L4A-AS2 expression was determined by qRT-PCR. (C) Western blot analysis indicated that EPB41L4A-AS2 reversed the EMT of NPC cells via repression of YBX1. (D) mRNA decay analyses showed that EPB41L4A-AS2 binds YBX1 to regulate Snail mRNA stability. (E) Western blot assays showed that EPB41L4A-AS2 binds YBX1 to reverse the EMT of NPC cells. (F)There is a negative relationship between Snail and EPB41L4A-AS2 in NPC tissues. *P < 0.05 and **P < 0.01.

Notably, the relevant literature on YBX1 showed that YBX1 could regulate the stability of Snail mRNA 29. As shown in Figures 7A and 7B, mRNA stability experiments found that overexpression of EBP41L4-AS2 reduced Snail mRNA stability while silencing EBP41L4-AS2 enhanced Snail mRNA stability. Co-transformation showed that EPB41L4A-AS2 regulates the stability of Snail mRNA by binding to YBX1 (Figure 7D). Silencing EBP41L4-AS2 promoted the EMT process of NPC cells, resulting in the promotion of EMT-related mesenchymal phenotype proteins (Snail, Vimentin, and N-cadherin) and decrease in the expression of epithelial associated proteins (E-cadherin). Contrastingly, the overexpression of EBP41L4-AS2 reversed the EMT process of NPC cells via repression of Snail (Figure 7C). Subsequently, changes in Vimentin and E-cadherin caused by EPB41L4A-AS2 depletion was partially reversed by YBX1 knockdown (Figure 7E). Clinical organization level shows a negative correlation between Snail and EPB41L4A-AS2 (Figure 7F).

### EPB41L4A-AS2 might serve as a competing endogenous RNA (ceRNA), sponging miR-107, inhibited the NPC cell invasion and migration

**Figure 8:**
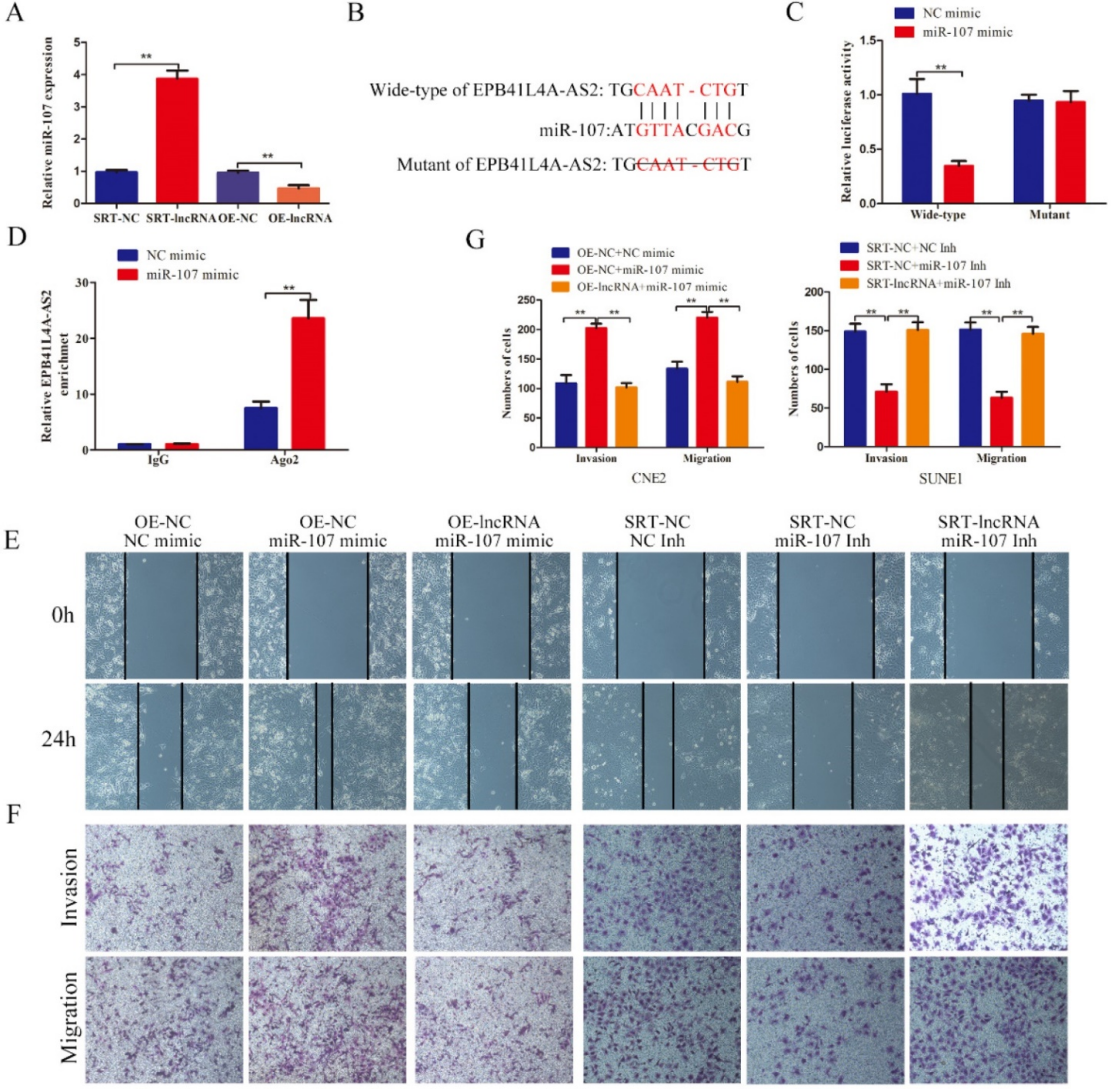
** EPB41L4A-AS2 might serve as a ceRNA, sponging miR-107, inhibited the invasion and migration of NPC cells.** (A) The expression of miR-107 in different treatment cells was detected by qRT-PCR. (B)The binding sequences between EPB41L4A-AS2 and miR-107. (C)The results of luciferase reporter showed that miR-107 directly targets EPB41L4A-AS2. (D) RIP assays exhibited that miR-107 overexpression significantly increased the enrichment of EPB41L4A-AS2 in the anti-Ago2 antibody. (E) The wound healing assays indicated that EPB41L4A-AS2 repressed the cell migration via targeting miR-107. (F and G) The transwell assays showed that EPB41L4A-AS2 inhibited the invasion and migration of NPC cells through repression of miR-107. *P < 0.05 and **P < 0.01.

Considering that EPB41L4A-AS2 was distributed not only in the nucleus but also in the cytoplasm, we deeply investigated the underlying mechanisms that the cytoplasm EPB41L4A-AS2 regulated the NPC tumor metastasis. Counting evidences have proved that lncRNAs serving as the sponges of miRNAs is the most common functional mechanisms that lncRNAs performs in the cytoplasm. We employed the bioinformatics analysis to look for the potential miRNAs that could bind to EPB41L4A-AS2. MiR-107 has caught our attention due to its high expression level associated with several advanced tumor progressions. Then we detected the expressions of miR-107 in NPC cells with different treatments and found that miR-107 expression was downregulated in NPC cells overexpressing EPB41L4A-AS2, while upregulated in the cells downregulating EPB41L4A-AS2 (Figure 8A). The correlation between EPB41L4A-AS2 and miR-107 was identified by luciferase reporter assay and RIP assays. The results of the luciferase reporter showed that miR-107 directly targets EPB41L4A-AS2 (Figures 8B and 8C). Moreover, RIP assays exhibited that miR-107 overexpression significantly increased the enrichment of EPB41L4A-AS2 in the anti-Ago2 antibody (Figure 8D).

In order to better characterize the correlation of EPB41L4A-AS2 and miR-107 in the progression of NPC tumor metastasis, we established the rescue biological experiments. The results indicated that EPB41L4A-AS2 overexpression retard the enhancement of the cell invasion and migration induced by miR-107 mimic, while EPB41L4A-AS2 knockdown antagonize the suppression caused by miR-107 inhibitor (Figures 8E-G). All these data suggests that EPB41L4A-AS2 might serve as a ceRNA, sponging miR-107 in NPC cells.

### EPB41L4A-AS2 blocked the invasion and migration of NPC cells by promoting LATS2 expression via sponging miR-107

**Figure 9:**
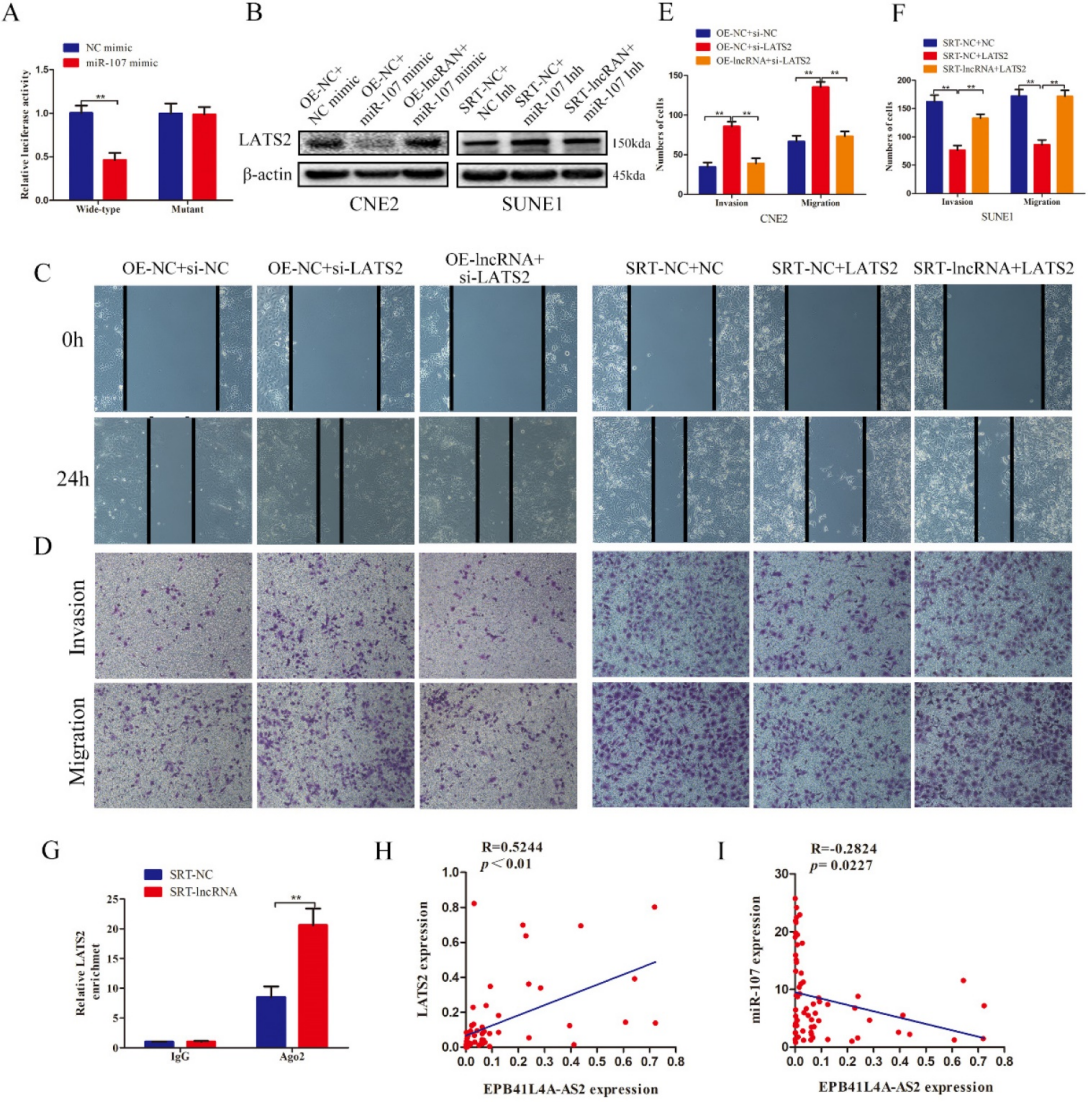
** EPB41L4A-AS2 blocked the invasion and migration of NPC cells by promoting LATS2 expression via sponging miR-107.** (A)The luciferase reporter assays identified that miR-107 directly targeted LATS2. (B) EPB41L4A-AS2 promoted the expression of LATS2 via sponging miR-107. (C) The results of wound healing assays indicated that EPB41L4A-AS2 blocked the migration of NPC cells via promoting LATS2 expressions. (D ,E and F) Transwell assays showed that EPB41L4A-AS2 reduced the cell invasion and migration of NPC through the promotion of LATS2. (G) RIP assays exhibited that EPB41L4A-AS2 knockdown significantly reduced the enrichment of LATS2 in the anti-Ago2 antibody. (H) There is a negative relationship between miR-107 and EPB41L4A-AS2. (I) There is a positive connection between LATS2 and EPB41L4A-AS2. *P < 0.05 and **P < 0.01.

The bioinformatics predictions, including Targetscan, Starbase and DIANA, were used to find out the target genes sharing the response elements of miR-107 with EPB41L4A-AS2. Among these results, we selected LATS2 for further investigation because previous work had proved that miR-107 directly targeted LATS2 in non-small cell lung cancer. In our study, our results confirmed that LATS2 was the direct target of miR-107 using the luciferase reporter assay (Figure 9A). We proceeded to explore whether EPB41L4A-AS2 coordinated the invasion and migration of NPC cells by promoting LATS2 expression via sponging miR-107. The results of western blot analyses indicated that EPB41L4-AS2 overexpression enhanced the expression of LATS2 which was repressed by miR-107 overexpression, while EPB41L4A-AS2 knockdown reversed the promotion of LATS2 induced by miR-107 down-regulation (Figure 9B). Herein, our rescue experiments showed that restoration of LATS2 reversed the EPB41L4A-AS2 knockdown-mediated promotion of the cell invasion and migration (Figure 9C-F). We also found that downregulated of LATS2 blocked the suppression of the invasion and migration of NPC cells induced by EPB41L4A-AS2 overexpression (Figure 9C-F). Meanwhile, the data of the RIP assays also proved that ectopic expression of EPB41L4A-AS2 in the Ago2-antibody reduced the enrichment of LATS2 (Figure 9G). We also employed qRT-PCR to detect the expressions of miR-107 and LATS2 in NPC tissues. The results indicated that there was a negative relationship between miR-107 and EPB41L4A-AS2, while a positive correlation between LATS2 and EPB41L4A-AS2 (Figures 9H and 9I). Taken together, our results revealed that EPB41L4A-AS2 blocked the invasion and migration of NPC cells by promoting LATS2 expression via sponging miR-107 in the cytoplasm.

## Discussion

Nasopharyngeal carcinoma (NPC) is notorious for its potentials to progress to the cervical lymph nodes and distant metastases at an early stage. Due to intensity-modulated radiation therapy (IMRT), the local control rate of NPC has greatly improved 30. However, locoregional control and prognosis are often not satisfactory due to the high rates of recurrence and metastasis. The incidence of distant metastasis of NPC varies from 7.7-20.3%, with a 5-year survival rate of about 50 to 60%. Besides, the recurrence rate of NPC remains between 10% and 40% after treatment 31. To date, the cause of metastasis and recurrence in NPC has not been thoroughly examined.

As the frequency of gene mutations and sites are rare in NPC, oncogenes, and tumor suppressor genes, have not been thoroughly researched 32. Recently, epigenetic changes have been found to play a crucial role in tumor development and progression 33, 34. LncRNA has higher tissue and cell-specific expression than protein-coding genes. Several classic lncRNAs have been validated in NPC, such as HOTAIR 35, MALAT1 36, CCAT1 37. Recognition of cancer-specific lncRNAs and their interacting molecules may be important for exploring new therapeutic targets, such as EPB41L4A-AS2, which is located in the genome 5q22.2 region. EPB41L4A-AS2 appears to be downregulated in lung 38, and liver cancer 39 inhibits proliferation and invasion and promotes apoptosis. EPB41L4A-AS2 is expressed at low levels in NPC cells and tissue. Moreover, its low expression is associated with the presence of distant metastasis and predicts adverse OS and PFS. The univariate and multivariate survival assays confirmed that EPB41L4A-AS2 expression was an independent predictor of PFS in patients with NPC. Taken together, it appears that the expression of EPB41L4A-AS2 might be a potential biomarker for patients with NPC.

Our research team previously found that lncRNA EPB41L4A-AS2 associated with TGF-β was involved in the metastasis of HNSCC 22. Here, we further investigated the association between the TGF-β signaling pathway and EPB41L4A-AS2. TGF-β is a pleiotropic cytokine that activates a series of signaling pathways that regulate cell proliferation, apoptosis, and metastasis, especially EMT, in many types of tumors 40. TGF-β signaling mediates EMT regression via classical SMAD and non-SMAD pathways, especially epithelial cell growth and plasticity. In typical TGF-β signaling, SMAD2 and SMAD3 are phosphorylated, while ligand binds to type 2 transmembrane receptor (TGFBR2) and recruits type 1 receptor (TGFBR1) and forms a complex with SMAD4 and subsequently nuclear translocation^41^, ^42^. This interacts with various transcriptional regulators or mediates target gene expression or suppression, inhibiting epithelial genes, and promoting mesenchymal protein expression. There is evidence that lncRNAs relate to the process of EMT and tumor metastasis. However, reports regarding TGF-β-associated lncRNAs in EMT are still limited. As mentioned before, our research team found that EPB41L4A-AS2 is a TGF-β-associated lncRNA that is negatively regulated by TGF-β signaling in a time- and dose-dependent manner. We employed the TGF-β receptor inhibitor, SB431542, to inhibit the phosphorylation of SMAD2. This found that EPB4AL4A-AS2, as the downstream effector of the TGF-β signaling pathway, was regulated through the classic SMAD pathway.

Our previous work verified that EPB41L4A-AS2 overexpression reversed the invasion and migration of TGF-β-induced EMT model cells. As a follow-up experiment, our team investigated the biological functions of EPB41L4A-AS2 in NPC carcinogenesis. Notably, the experiments showed that EPB41L4A-AS2 overexpression displayed no significant positive or negative effects on cell proliferation and cell cycle. However, ectopic overexpression of EPB41L4A-AS2 inhibited SUNE1 invasion and metastasis, indicating that EPB41L4A-AS2 might function as a metastasis-associated gene in NPC development. We found that low expression of EPB41L4A-AS2 was related to adverse survival outcomes in NPC. Nevertheless, the precise mechanism by which EPB41L4A-AS2 is responsible for NPC metastasis in promoting EMT remains to be elucidated.

The subcellular fractionation location assays indicated that EPB41L4A-AS2 was both distributed in the nucleus and cytoplasm. Subsequently, we carried out RNA pulldown experiments to examine EPB41L4A-AS2-associated proteins and performed a mass spectrometry analysis of the binding proteins. There were many potential binding proteins identified in the mass spectrometry analysis, such as ANXA2, GAPDH, and YBX1. Among them, YBX1 attracted the attention of our research group, and it has been widely investigated by our laboratory. Our previous work showed that LINC01133 inhibited the EMT, cell invasion, and metastasis of NPC by directly binding to YBX1. YBX-1 belongs to DNA/RNA binding proteins and has an evolutionarily conserved cold-shock domain (CSD)^14^. In particular, YBX1 binds to the sequence 5'-CTGATTGG-3' of Y-box (present on the promoter of many genes) to regulate gene expressions, such as genes encoding the EGF receptor, multidrug resistance protein, and cell division cycle protein^43^. Functionally, YBX1 plays an important role in a number of cellular functions, such as transcriptional regulation, mRNA splicing, and stress response to extracellular signals 14. Critically, YBX1 is considered to be an oncoprotein that is up-regulated in many malignant cancers, promotes malignant transformation (proliferation, invasion, metastasis) and angiogenesis of various cancers as well as genomic instability. It is closely related to chemotherapy drug resistance and poor prognosis. In a recent study, tRNA binding to YBX1 was found in oncogenic transcript destabilization and was shown to mediate enhanced expression. Moreover, YBX1 has been found to promote epithelial-mesenchymal transition by coordinating EMT-related transcription factor expression networks and acting together to promote metastasis and diffusion in multiple studies 44. Collectively, the YBX1-mediated tumor metastasis regulation network may be a universal target for the treatment of various tumors. In this study, we delineated the mechanism by which TGF-β inhibits EPB41L4A-AS2 in NPC, which showed that EPB41L4A-AS2 was reactivated after we silenced key genes in the TGF-β molecule pathway. Next, for the molecular mechanism of EPB41L4A-AS2, we identified YBX1, which binds and interacts with EPB41L4A-AS2.

Notably, a literature study found that YBX1 directly activates Snail mRNA independent of cap translation, which is related to the downregulation of epithelial markers in EMT and the activation of mesenchymal markers 45. We observed whether EPB41L4A-AS2 affected the stability of Snail mRNA after silenced or overexpressed YBX1. Surprisingly, the reduction of EPB41L4A-AS2 enhanced the stability of Snail mRNA, while an overexpression of EBP41L4-AS2 had the opposite effect in nasopharyngeal carcinoma cells. Further investment showed that EPB41L4A-AS2 knockdwon affected the subcellular location of YBX1, which might be the method that EPB41L4A-AS2 affected Snail mRNA stability. Based on these findings, we concluded that EPB41L4A-AS2 relies on binding to YBX1 to regulate Snail expression in the nucleus. However, affecting Snail mRNA stability was one of the mechanism performed by YBX1, more work needs to be done to explore the relationship among EBP41L4A-AS2, YBX1 and their downstream targets. Snail belongs to the zinc finger family and is one of the earliest found transcription factors. It inhibits E-cadherin transcription via directly binding to the specific E-box of the E-cadherin promoter 46. For example, EZH2 inhibits E-cadherin and promotes EMT by interacting with HDAC1/HDAC2/Snail to form a co-repressor complex 47. Another study on up-regulated Bmi-1 found that it stabilized Snail-induced EMT and enhanced the motility and invasiveness of NPC cells by regulating PI3K/Akt/GSK-3b signaling 48. EIF4E promotes NPC cell invasion by combining Snail mRNA for translation initiation 49. Consistently, the hypermethylation of HOPX in the promoter region mediates epigenetic silencing of Snail transcription to inhibit metastasis and EMT and enhance the chemical sensitivity of NPC cells 45. Recent studies regarding the role of Snail in NPC have shown that Snail mediates invasion and metastasis of NPC mainly through epithelial-mesenchymal transition. In this study, our data proved that EPB41L4A-AS2 reduced the expression of Snail to reverse the EMT progression via directly binding to YBX1.

Growing evidence has shown that cytoplasmic lncRNAs usually serve as as the sponges of miRNAs to release the expressions of these targets which were repressed by miRNAs. For example, LINC00152 drive ANXA2-mediated invasion and metastasis of NPC cells via sponging miR-613 19. Another lncRNA ZNRD1-AS1, up-regulated in NPC and associated with advanced TNM stages, was also reported increased the NPC cell invasiveness and metastasis via affecting miR-335-ROCK1 axis 21. In some other cancers, Wang and colleagues showed that the EPB41L4A-AS2-miR-301a-5p-FOXL1 axis inhibits hepatocellular carcinoma development 39. In ovarian cancer, researchers found that EPB41L4A-AS2 reversed the miR-103a-mediated tumor-promoting effects on the cell viability and metastasis via competing for the shared elements 50. In this study, we found EPB41L4A-AS2 served as a competing endogenous RNA (ceRNA), sponging miR-107 and promoting the invasion and metastasis of NPC cells. miR-107, as a famous miRNA, has been reported in many cancers. The relationship between miR-107 and lncRNAs was also explored by many researchers. Han et al reported that lncRNA MIR503HG reversed the tumorigenesis of cervical cancer via repressing the expression of miR-107 17, while another report showed lncRNA LINC00467 contributes to tumorigenesis of cervical cancer cells through targeting miR-107 18. In view of these findings, the role of miR-107 in different cancers might perform carcinogenicity or cancer-suppressor functions. In our study, we established the rescue experiment models and found that overexpression of miR-107 antagonized the suppressible effects induced by EPB41L4A-AS2, indicating miR-107 functioned as tumor-promoter in NPC development.

To further characterize the role of EPB41L4A-AS2-miR-107 axis in NPC metastasis, the downstream regulatory mechanism was explored. In non-small cell lung cancer (NSCLC), Jin et al. reported miR-107 targeted LATS2 to suppress the metastasis of NSCLC cells 51. During our study, we also detected the expression of LATS2 in EPB41L4A-AS2 overexpression or knockown cells. The data indicated that upregulated expression of EPB41L4A-AS2 promoted the LATS2 expression, while downregulated expression of EPB41L4A-AS2 reduced the LATS2 expression. Further investigation showed that EPB41L4A-AS2 increased the expression level of LATS2 via sponging miR-107. Previous work has demonstrated that LATS2 namely large tumor suppressor kinase 2 52, functions as a tumor suppressor effector, which has been reported in many cancers and its low expression was linked with advanced tumor progression. For instance, miR-650 directly targets and inhibits the expression of LATS2 to promote the metastasis and epithelial-mesenchymal transition of hepatocellular carcinoma cells 53. In Gallbladder cancer, lncRNA MEG3 performs functions by regulating the stability of EZH2 to regulate the downstream target gene LATS2 and thereby inhibits invasion and proliferation 54. In short, different signaling pathways inhibit the transcription and translation of LATS2 or the pathway regulated by LATS2 to exert tumor suppressor effect in various cancers.

## Conclusions

**Figure 10:**
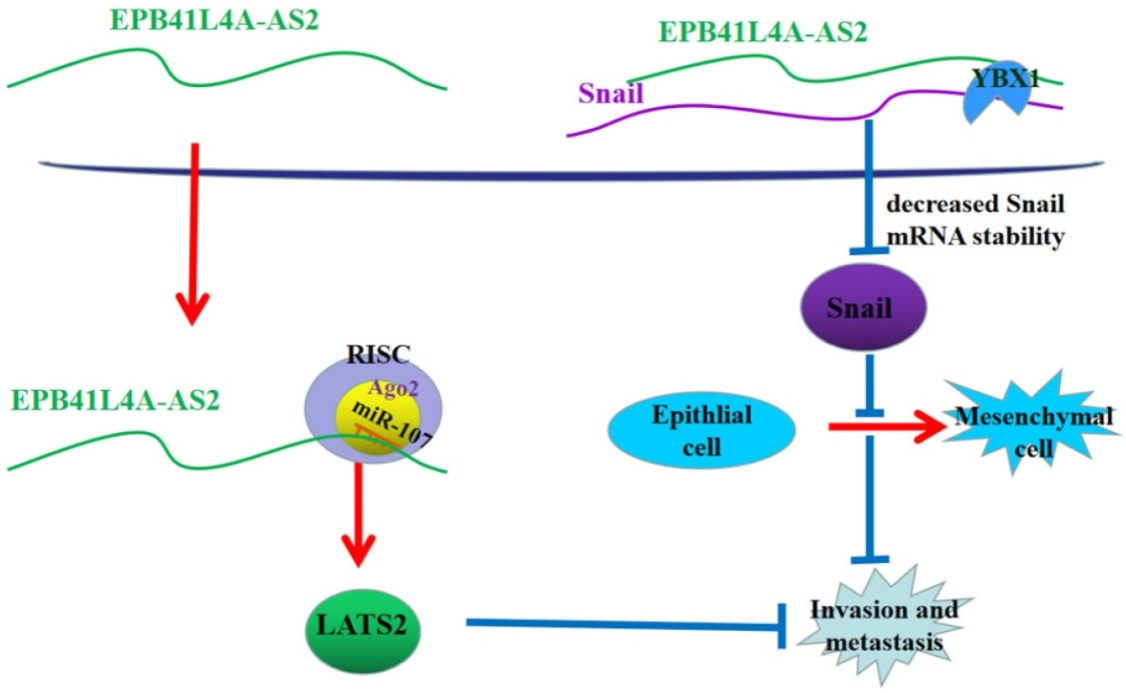
** A proposed mode for EPB41L4A-AS2-mediated the regulation of NPC metastasis.** In the nucleus, EPB41L4A-AS2 relies on binding YBX1 to enhance the stability of snail mRNA to reduce the expression of E-cadherin and promote EMT. In the cytoplasm, EPB41L4A-AS2 suppresses NPC metastasis via mir-107-LATS2 axis at the post-transcriptional level.

In conclusion, in the nucleus, EPB41L4A-AS2 relies on binding YBX1 to reduce the stability of Snail mRNA to promote the expression of E-cadherin and reverse EMT. In the cytoplasm, EPB41L4A-AS2 suppresses NPC metastasis via miR-107-LATS2 axis at the post-transcriptional level (Figure 10). The above two approaches jointly inhibit the metastasis of nasopharyngeal carcinoma. The findings of this study help to further understand the metastasis mechanism of NPC and could help in the prevention and treatment of NPC metastasis.

## Supplementary Material

Supplementary figures.

## Figures and Tables

**Table 1:** Correlation between EPB41L4A-AS2 expression and clinical characteristics of NPC patients

Characteristics	No of patients (n=65)	EPB41L4A-AS2 High group	EPB41L4A-AS2 Low group	*P*-value
**Age (years)**				
≤51	33	18	15	0.536
>51	32	15	17	
**Gender**				
female	17	8	9	0.722
male	48	25	23	
**TNM stage**				
II-III	27	17	10	0.097
IV	38	16	22	
**T classification**				
T1-T2	17	11	6	0.181
T3-T4	48	22	26	
**N classification**				
N0-N1	31	20	11	0.048
N2-N3	34	13	21	
**M classification**				
M0	51	27	24	0.504
M1	14	6	8	

**Table 2:** Prognostic factors for OS and PFS from univariate analysis

Characteristics	No.	OS		PFS	
		HR (95%CI)	*p*	HR (95%CI)	*p*
**Age (years)**					
≤51	33	1		1	
>51	32	1.430 (0.382-5.357)	0.596	0.952 (0.356-2.541)	0.921
**Gender**					
Female	17	1		1	
Male	48	2.995 (0.372-24.124)	0.303	1.115 (0.349-3.557)	0.854
**TMN stage**					
II-III	27	1		1	
IV	38	2.323 (0.553-9.753)	0.249	1.221 (0.450-3.311)	0.695
**T classification**					
T1-2	17	1		1	
T3-4	48	0.998 (0.246-4.044)	0.998	0.861 (0.307-2.415)	0.776
**N classification**					
N0-1	31	1		1	
N2-3	34	7.315 (0.910-58.798)	0.061	1.369 (0.504-3.716)	0.538
**M classification**					
M0	51	1		1	
M1	14	2.417 (0.436-1.398)	0.312	4.203 (1.405-12.578)	0.10
**EPB41L4A-AS2 expression**					
High	33	1		1	
Low	32	5.687 (1.137-28.458)	0.034	3.809 (1.130-12.832)	0.031

**Table 3:** Prognostic factors for OS and PFS from multivariate analysis

Characteristics	No65.	OS		PFS	
		HR (95%CI)	*p*	HR (95%CI)	*p*
**Age (years)**					
≤51	33	1		1	
>51	32	0.804 (0.150-4.305)	0.798	0.475 (0.135-1.677)	0.248
**Gender**					
Female	17	1		1	
Male	48	2.745 (0.232-32.452)	0.423	1.361 (0.354-5.230)	0.654
**TMN stage**					
II-III	27	1		1	
IV	38	0.743 (0.085-6.516)	0.788	0.711 (0.217-2.328)	0.573
**T stage**					
T1-2	17	1		1	
T3-4	48	0.931 (0.175-4.938)	0.933	0.818 (0.236-2.838)	0.752
**N stage**					
N0-1	31	1		1	
N2-3	34	7.967 (0.513-123.759)	0.138	0.999 (0.308-3.237)	0.998
**M stage**					
M0	51	1		1	
M1	14	2.288 (0.312-16.787)	0.416	5.766 (1.618-20.549)	0.007
**EPB41L4A-AS2 expression**					
High	33	1		1	
Low	32	8.121 (0.862-76.469)	0.067	5.227 (1.297-21.065)	0.002

## Abbreviations

NPC: nasopharyngeal carcinoma
EBV: Epstein-Barr virus
LncRNA: long noncoding RNA
EMT: epithelial-to-mesenchymal transition
TGF-β: transforming growth factor-beta
LATS2: namely large tumor suppressor kinase 2
NS: not significant
