# Supplementary material for: LncRNA EPB41L4A-AS2 represses Nasopharyngeal Carcinoma Metastasis by binding to YBX1 in the Nucleus and Sponging MiR-107 in the Cytoplasm
Source: Int J Biol Sci. 2021 May 11;17(8):1963–78. doi: 10.7150/ijbs.55557 (PMC8193272; doi:10.7150/ijbs.55557)
Supplement: Supplementary file 1 — Supplementary figures. [file ijbsv17p1963s1.pdf]

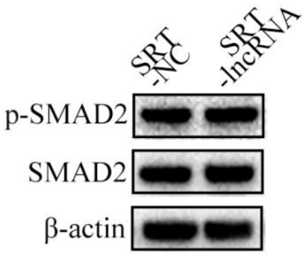

Supplementary Figure 1

Western blot assays indicated that EPB41L4A-AS2 knockdown showed no obvious effects on the expressions of SMAD and p-SMAD2.

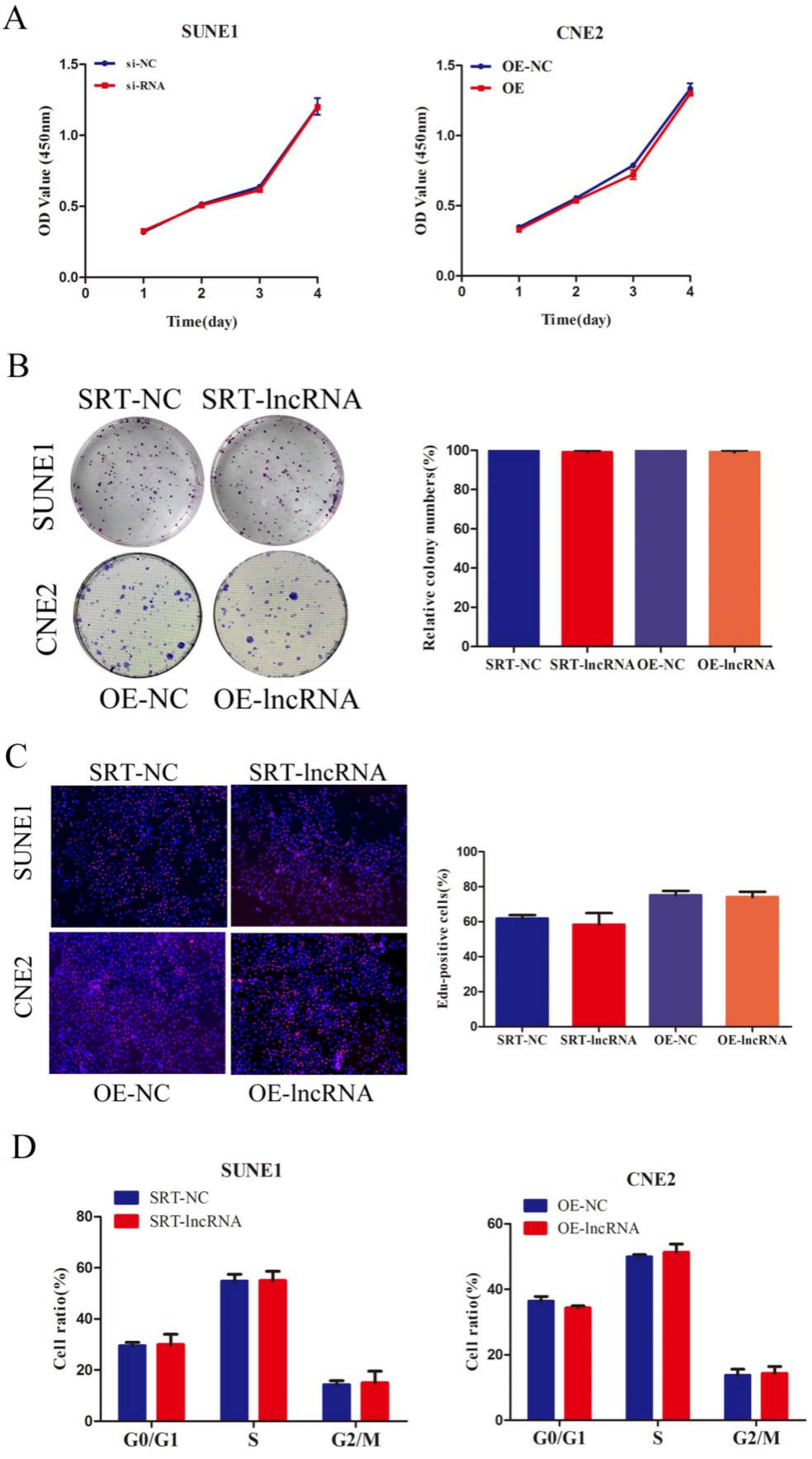

Supplementary Figure 2

(A-C) CCK8, colony formation, and EDU assays were used to explore the cell viability of NPC cells under different treatments. (D) The cell cycle was detected by flow cytometry.

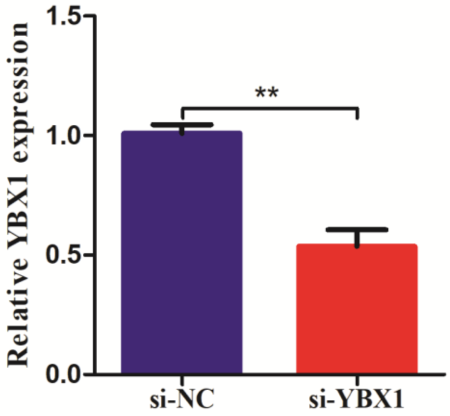

Supplementary Figure 3

The results indiated that siRNA against YBX1 effectively reduced YBX1 expressions.

**P* < 0.05 and ***P* < 0.01.
